# Role of outer dense fiber of sperm tails 2-like (ODF2L) protein in ciliation in mammalian cells and in zebrafish

**DOI:** 10.1186/2046-2530-4-S1-P32

**Published:** 2015-07-13

**Authors:** P De Saram, C Wilkinson, J Murdoch

**Affiliations:** 1 Biological Sciences, Royal Holloway University of London, Egham, UK

## Background

The centrosome is a subcellular organelle which plays an important role within the cell as the microtubule organising centre (MTOC) and many other cellular functions. In a quiescent cell, centrioles can migrate to the apical surface of the cell and nucleate hair-like projections called cilia and flagella. The stages of ciliogenesis require a large number of proteins and complexes to be trafficked through to the cilium and are carefully coordinated by the centrosome and centrosome-associated proteins. Here, we studied centrosome proteins named outer dense fiber-2 like (ODF2L) to establish their role in ciliogenesis *in vitro *and in zebrafish (*Danio rerio*).

## Results

We identified the ODF2L as a satellite protein in proliferating cells by using immunofluorescent-labeling and expression of this protein is changed when the ciliation is induced. When ciliation induced, ODF2L seems to localise into the centrioles or completely diminish the expression in hTERT-RPE-1 cells. When lysate of the quiescent cells was analysed for the ODF2L expression revealed the complete removal of the protein. When ODF2L was over expressed, hTERT-RPE-1, were unable to ciliate even after initiation of ciliation by serum depravation. Furthermore, knocking down of ODF2L with SiRNA in hTERT-RPE-1 cells resulted in four times more cilia cultured in serum supplemented media. In zebrafish depleting the transcript by using anti-sensing morpholinos resulted in the phenotype of varied development and curved back with ectopic otoliths which resembles other ciliary function compromised phenotypes observed when the known ciliary components were depleted. Furthermore, whole mount immunoflorescent studies of morphants revealed shortened cilia in the pronephros.

## Conclusion

ODF2L may negatively regulate ciliation by changing the expression and its localisation within mammalian cells however in zebrafish exact function is yet to be discovered.

